# Dawn of diverse shelled and carbonaceous animal microfossils at ~ 571 Ma

**DOI:** 10.1038/s41598-024-65671-4

**Published:** 2024-06-28

**Authors:** Luana Morais, Bernardo T. Freitas, Thomas Rich Fairchild, Rolando Esteban Clavijo Arcos, Marcel Guillong, Derek Vance, Marcelo Da Roz de Campos, Marly Babinski, Luiz Gustavo Pereira, Juliana M. Leme, Paulo C. Boggiani, Gabriel L. Osés, Isaac D. Rudnitzki, Douglas Galante, Fabio Rodrigues, Ricardo I. F. Trindade

**Affiliations:** 1 Department of Geophysics, Institute of Astronomy, Geophysics and Atmospheric Sciences, University of São Paulo (USP), São Paulo, SP Brazil; 2 Universidade Estadual de Campinas (UNICAMP), Campinas, SP Brazil; 3 Institute of Geosciences, University of São Paulo (USP), São Paulo, SP Brazil; 4 Institute of Geochemistry and Petrology, Department of Earth Sciences, ETH Zurich, Clausiusstrasse 25, 8092 Zurich, Switzerland; 5 Programa de Pós-Doutorado, Instituto de Física, Universidade de São Paulo (USP), Rua do Matão, 1371, São Paulo, 05508090 Brazil; 6 Laboratório de Arqueometria e Ciências Aplicadas ao Patrimônio Cultural, Instituto de Física, Universidade de São Paulo (USP), Rua do Matão, 1371, São Paulo, 05508090 Brazil; 7 Departament of Geology, Federal University of Ouro Preto (UFOP), Ouro Preto, MG Brazil; 8 Laboratório Nacional de Luz Síncrotron, Campinas, SP Brazil; 9 Departamento de Química Fundamental, Instituto de Química, Universidade de São Paulo (USP), São Paulo, SP Brazil; 10 Present Address: Department of Geology, São Paulo State University (UNESP), Rio Claro, 13506-900 Brazil

**Keywords:** Palaeoecology, Palaeoecology

## Abstract

The Ediacaran-Cambrian transition documents a critical stage in the diversification of animals. The global fossil record documents the appearance of cloudinomorphs and other shelled tubular organisms followed by non-biomineralized small carbonaceous fossils and by the highly diversified small shelly fossils between ~ 550 and 530 Ma. Here, we report diverse microfossils in thin sections and hand samples from the Ediacaran Bocaina Formation, Brazil, separated into five descriptive categories: elongate solid structures (ES); elongate filled structures (EF); two types of equidimensional structures (EQ 1 and 2) and elongate hollow structures with coiled ends (CE). These specimens, interpreted as diversified candidate metazoans, predate the latest Ediacaran biomineralized index macrofossils of the *Cloudina-Corumbella-Namacalathus* biozone in the overlying Tamengo Formation. Our new carbonate U–Pb ages for the Bocaina Formation, position this novel fossil record at 571 ± 9 Ma (weighted mean age). Thus, our data point to diversification of metazoans, including biomineralized specimens reminiscent of sections of cloudinids, protoconodonts, anabaritids, and hyolithids, in addition to organo-phosphatic surficial coverings of animals, demonstrably earlier than the record of the earliest known skeletonized metazoan fossils.

## Introduction

The Ediacaran to Cambrian transition (~ 550–530 Ma) is recognized as one of the most transformative moments in Earth history, marked by the ‘explosion’ of animals, including non-biomineralizing^1^ and biomineralized organisms^2,3^. Small carbonaceous fossils are sub-millimetric fragile non-biomineralized remains, commonly interpreted as the surficial covering of several animals, such as crustaceans, molluscs, annelids and priapulids^4–7^, in addition to taxa with uncertain affinities^8^.

Biomineralization is one of the major evolutionary innovations in the history of life, evolved in many clades, changing the functional biology of innumerable protists, plants, and animals^9–11^. This innovation was already well established in unicellular eukaryotes by the Tonian (~ 811–740 Ma)^12,13^ and tentatively ascribed to sponges during the Cryogenian (~ 720–635 Ma)^14–18^. Well-established and widespread biomineralization in animals, mainly represented by the extinct Nama assemblage of *Cloudina*, *Sinotubulites*, *Namacalathus, Corumbella* and Namapoikia^19–21^, is currently recognized as having started in the latest Ediacaran, between 550 and 539 Ma^2,3^.

Above the Ediacaran-Cambrian boundary, between 539 and 510 Ma^3^, the fossil assemblage commonly becomes dominated by diversified submillimeter to millimeter-scale shells and sclerites, interpreted as remains of modern metazoan biomineralizers, such as molluscs, echinoderms and brachiopods^22,23^, as well as taxa with uncertain affinities^16^. The diversity and disparity of biomineralizers have been related to increasing complexity in ecosystems^16,24,25^. The appearance of diverse groups capable of biomineralization, suggests development of distinct modes of biomineralization^3^. These innovations, likely causes and consequences of environmental changes during the Late Ediacaran and Cambrian, are often assigned to the Cambrian biomineralization event^26,27^. Diversified shells and sclerites in Lower Cambrian strata are commonly referred to as small shelly fossils and include many forms used as biostratigraphic tools globally^16,22, 25, 28–30^.

The apparent collapse of the Nama Biota at the end of the Ediacaran Period^31^ and the sudden appearance of small shelly fossils in lower Cambrian deposits^25^ has been ascribed to major environmental changes and extinction of Ediacaran metazoans, separating the Proterozoic and Phanerozoic Eons at 538 Ma^25,32–34^. However, recent discoveries indicative or suggestive of overlapping fossil assemblages that were once considered restricted to specific time intervals (Yang et al.^35^, Refs.^20,24, 36–40^), may require alternative interpretations for the fossil record along the Ediacaran-Cambrian transition^20,38,41,42^.

Here, we report diverse microfossils from Ediacaran deposits of the Bocaina Formation, underlying the Nama Biota-bearing deposits of the latest Ediacaran Tamengo Formation, both units from the Corumbá Group, Brazil. The novel fossils are (i) positioned within an updated detailed stratigraphic context, including new cloudinomorph occurrences in the Tamengo Formation along the South Paraguay Belt; (ii) constrained by new geochronological data; and (iii) compared to Neoproterozoic and Cambrian non-biomineralizing and biomineralizing organisms. With these means we not only report a new diversified fossil assemblage displaying biological traits tens of millions of years before their broad establishment in the fossil record, but also reveal the biased nature of the Ediacaran to Cambrian fossil succession.

###  Geological setting

**Figure 1:**
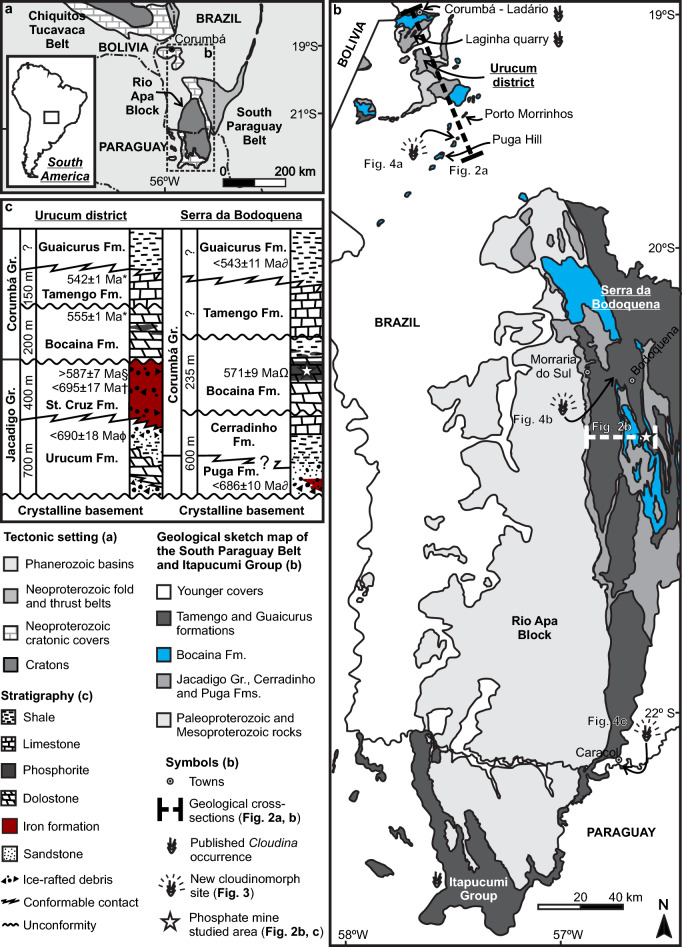
Geological setting of the study area. (**a**) Regional tectonic context. (**b**) Sketch map of South Paraguay Belt and Itapucumi Group. (**c**) Neoproterozoic stratigraphy in the Urucum district and Serra da Bodoquena *Parry et al.^48^, ^§^Piacentini et al.^49^; ^†^Frei et al.^50^; ^ϕ^Freitas et al.^51^; ^∂^McGee et al.^52^; ^Ω^This work. Geology after Campanha et al.^44^; Romero et al.^53^, McGee et al. 2018; Warren et al.^54^; Freitas et al.^51^, Hippert et al.^47^. Figure created using Corel X8.

**Figure 2:**
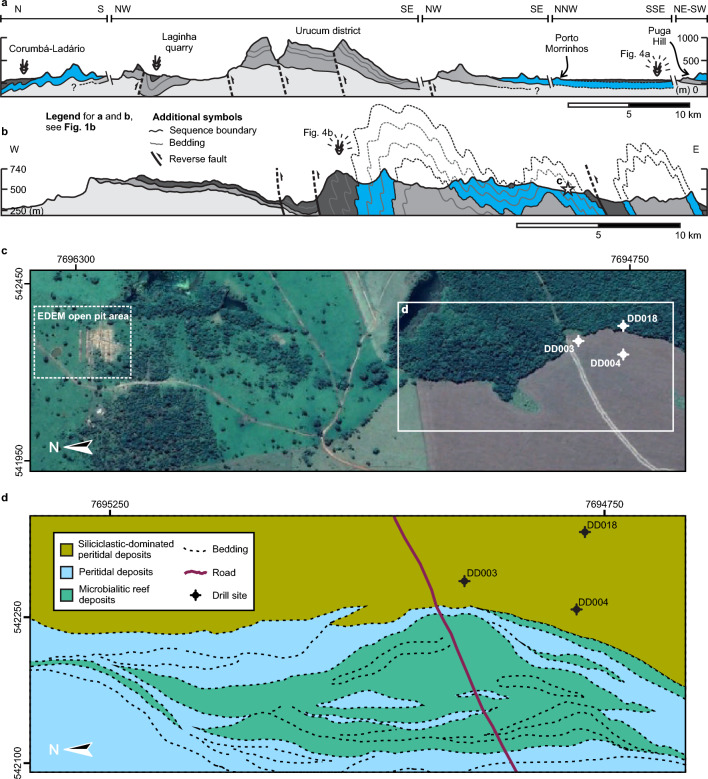
Regional to local geological setting. (**a**,**b**) Geological cross-sections of the Urucum district (**a**) and Serra da Bodoquena (**b**) depicting updated regional stratigraphy and structural geology of the South Paraguay Belt. (**c**) Satellite image available from Google Earth displaying the positions of the studied cores (DD003, DD004 and DD018) and EDEM open pit mine area. Google Earth Pro V 7.3.6.9345 (October, 2018). Bonito-MS, Brazil. 20º 50′ 29.07″ S, 56º 35′ 53.21″ W, Eye alt 2.7 km. CNES/Airbus 2023. http://www.earth.google.com [January 24, 2023]. (**d**) Detailed surface geology nearby the drill sites of the studied cores and open pit mine, revealing the reef geometry of associated microbialites and breccias. Vertical exaggeration of reef geometry due to 30º to 60º dips towards top of page (East) and truncation by sub-horizontal topography.

The Bocaina Formation occurs around the Urucum district in Corumbá and in the Serra da Bodoquena, Southern Paraguay Belt, SW-Brazil (Figs. 1, 2)^43,44^. In both regions, the Bocaina Formation is virtually unmetamorphosed. It forms part of the carbonate-dominated succession of the Corumbá Group, consisting of dolomitic grainstones and stromatolitic deposits with shale locally present in its upper part^43,45–47^ (Figs. 1, 2). Significant bedded and nodular phosphate deposits occur at intermediate to upper stratigraphic positions, as well as phosphorite clast accumulations^43,45–47^.

Truncated by regional erosional unconformities at both its top^43,44,55^ and base^44,47, 53^, this work), the Bocaina Formation overlies Paleoproterozoic crystalline basement and Cryogenian to Early Ediacaran post-glacial deposits^44,47, 51, 53^ (Figs. 1, 2a,b). The maximum thickness of the Bocaina Formation is estimated to be from one to a few hundred meters^43^. A complete measured stratigraphic section is not available for the unit. Nevertheless, maximum thicknesses between 140 and 235 m for the formation are evident in cross-sections perpendicular to strike and on the flanks of simpler folds in the Serra da Bodoquena^44^.

The Bocaina Formation successions are overlain by the limestone-dominated Tamengo Formation^43,44, 55^, which has a maximum measured stratigraphic thickness of around 150 m near the Urucum district^56^. The Tamengo Formation is known worldwide for its abundance of the late Ediacaran skeletal macrofossils *Cloudina* and *Corumbella*. Both have been found within Corumbá and adjacent Ladário in Brazil^43,57,58^ (Figs. 1, 2a) and hundreds of kilometers to the south in Paraguay^59^ (Fig. 1b), but not yet in the Serra da Bodoquena.

Until now, geochronological constraints on the age of these formations have been restricted to the Urucum district and surroundings (Fig. 1). Zircon grains, previously interpreted as related to ash beds within the Tamengo Formation^56,48, 58^ in Corumbá yield weighted mean ages of 541.8 ± 1 Ma and 542.4 ± 0.7 Ma (U–Pb, CA-TIMS^48^,). Similarly, zircon grains retrieved from an outcrop of the Bocaina Formation at Porto Morrinhos (Figs. 1, 2a), a few meters above phosphorite intercalations in a stromatolite-dominated succession, were dated at 555.2 ± 0.7 Ma (U–Pb, CA-TIMS; Ref.^48^).

Correlation of the latest Ediacaran *Cloudina*-bearing deposits at Corumbá and in Paraguay with the uppermost limestones of the Corumbá Group in Serra da Bodoquena is based on lithostratigraphy^43,44, 55^ and supported by carbon and strontium isotope data^43,54, 56,60,61^. The dolomitic successions and phosphate-rich horizons of the Bocaina Formation have thus been considered to predate the ca. 550 Ma carbon isotope excursion registered in the Tamengo limestones and equivalent deposits throughout the South Paraguay Belt^33,56^.

**Figure 3:**
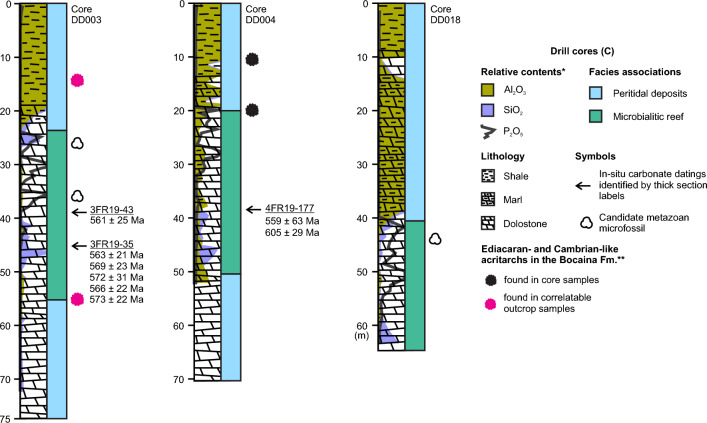
Columnar sections from studied drill cores. The entire succession represented in the three cores occurs within the Bocaina Formation, above the basal sequence boundary separating this unit from the deposits of the Puga and Cerradinho formations, and below the regional unconformity defining the top of the Bocaina Formation and the base of the Tamengo Formation (Fig. 2b). Note positions of fossils and dated samples, as well as lithological variations and interpreted facies associations through drill cores ALR2-DD003, ALR2-DD004, and ALR2-DD018. *Relative proportions of Al, Si and P compared to other major chemical elements present along the surveyed lithologies based on chemical analyses carried out by EDEM. **Previously published results^46^. For detailed geological setting and location of drill sites, see Fig. 2c,d.

Recent micropaleontological investigation of the Bocaina Formation in the studied phosphate mine area (Figs. 1, 2) revealed diverse acritarchs similar to one Cambrian genus and other taxa described in the Doushantuo-Pertatataka assemblage (Fig. 3)^46^, an assemblage first recognized in the < 580 Ma Pertatataka Formation, Australia^62^, and the c. 609 to 570 Ma Doushantuo Formation (Weng’an biota), China^63^.

## Materials and methods

**Figure 4:**
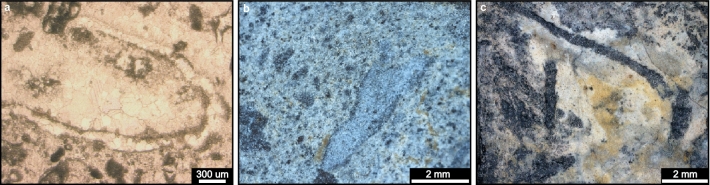
New occurrences of cloudinomorphs in limestones of the Tamengo Formation. (**a**) Photomicrograph (cross-section view) of U-shaped specimen constricted near the base from a bed of fossiliferous calcitic rudstone (thin section SM01GA, 19° 32′ 50.96″ S, 57° 27′ 29.42″ W, between Porto Morrinhos and Puga Hill). (**b**) Plan view of limestone bedding exhibiting poorly-preserved V-shaped specimen that expands slightly towards its open apex (sample 22BD21-A, 20º 32′ 37.86″ S, 56º 44′ 24.29″ W, ~ 6.5 km W from Bodoquena in the direction of Morraria do Sul). (**c**) Plan view of limestone bedding showing well-preserved tubular specimens exhibiting overlapping, ‘funnel-in-funnel’ construction, typical of the genus *Cloudina* (sample AP-01E, 22º 13′ 59.31″ S, 56º 47′ 45.62″ W, ~ 4 km straight to SE from Caracol).

**Figure 5:**
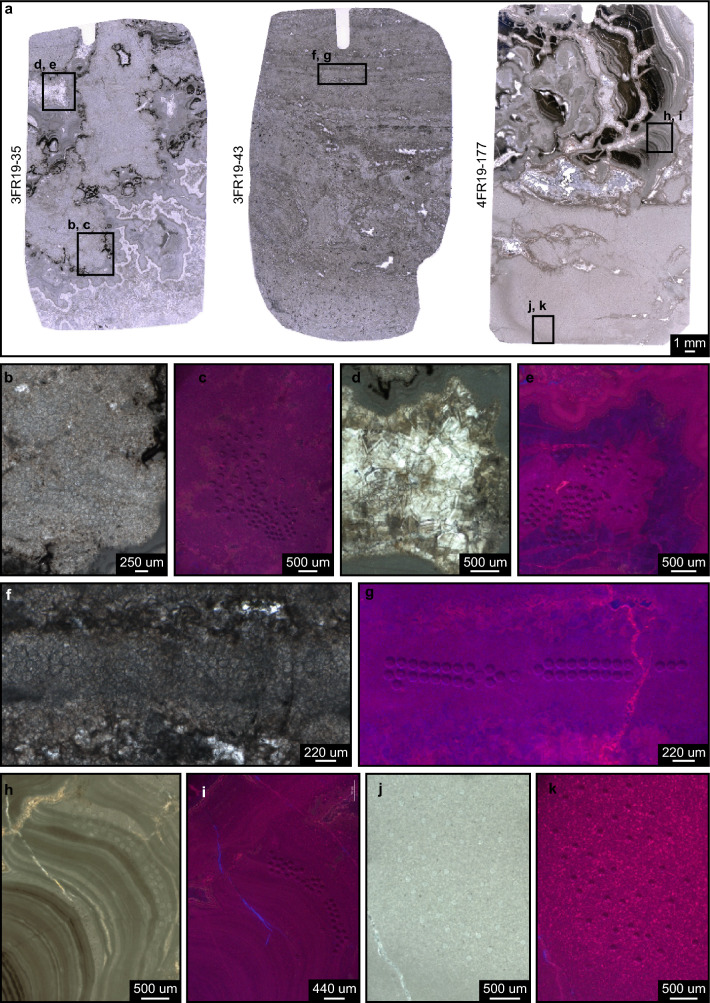
Dated carbonate microfacies. (**a**) Analyzed thick sections including: a thrombolitic feature associated with stromatolites and vugs (left); domal to stratiform stromatolitic microfacies (center); and fine-grained, possibly thrombolitic microfacies passing upward to bulbose and columnar stromatolites (right). (**b**–**k**) Cross-polarized and CL images, respectively, of the lower part of a thrombolite (**b**,**c**); a vug (**d**,**e**); stratiform stromatolite laminae (**f**,**g**); columnar stromatolitic laminae (**h**,**i**); fine grained, possibly thrombolitic microfacies (**j**,**k**). The circular spots correspond to ablated areas for U–Pb analysis. The larger spots in c are ablated areas for future studies.

**Figure 6:**
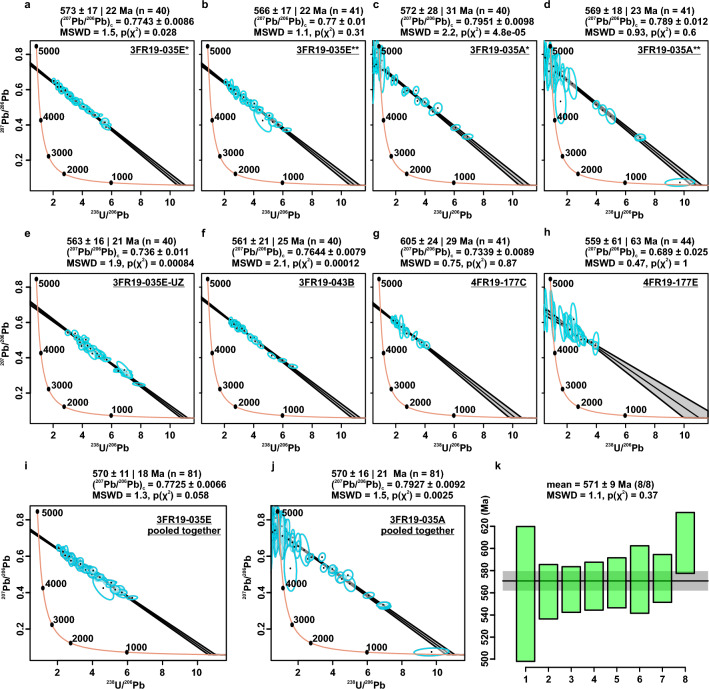
Carbonate geochronology results. Tera-Wasserburg diagrams of data obtained from in-situ U–Pb LA-ICP-MS of the lower part of a thrombolitic feature (**a**,**b**) (Fig. 5a–c); early cements in a vug (**c**,**d**) (Fig. 5d,e); the upper part of the thrombolitic feature (**e**); stratiform stromatolite (**f**) (Fig. 5f,g); stromatolitic laminae in small column (**g**) (Fig. 5h,i); and fine-grained microfacies possibly thrombolitic (**h**) (Fig. 5j,k). (**i**) Data in (**a**) and (**b**) pooled together. (**j**) Data in (**c**) and (**d**) pooled together. (**k**) Weighted mean age of the eight dated areas from the three analyzed samples (Fig. 5a). All ellipses are 2σ uncertainties. Age uncertainties are displayed as 2σ/2σ propagated systematic uncertainties.

Microfossils found within the Bocaina Formation were observed in samples collected from drill cores and an open pit phosphate mine in the study area in the Serra da Bodoquena (Figs. 1, 2). The drilled and mined carbonate deposits were subjected to sedimentary logging and facies analysis and positioned within a detailed geological setting (Figs. 1, 2 and 3). The updated stratigraphy of the South Paraguay Belt is reinforced by the report of new geographical occurrences of cloudinomorphs in the overlying Tamengo Formation (Figs. 1, 2a,b, 4) and by new age determinations of carbonates from the studied drill cores of the Bocaina Formation in the Serra da Bodoquena (Figs. 1, 2, 3, 5 and 6; Supplementary data, Tables 1–4). Fossiliferous samples and thin sections are stored in the collections of the Laboratório de Paleontologia Sistemática (LPS—Laboratory of Systematic Paleontology), Universidade de São Paulo, Brazil.

**Figure 7:**
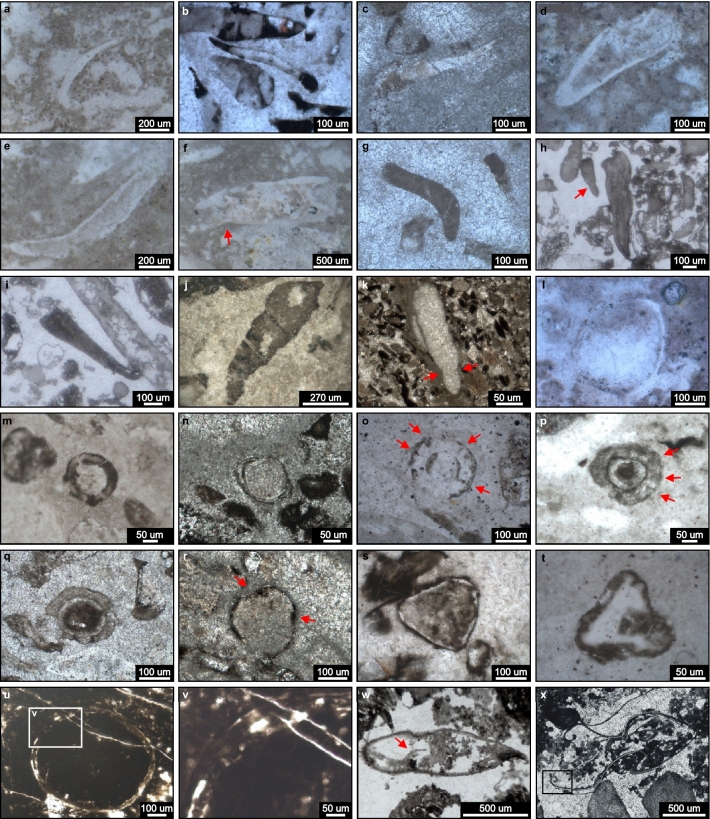
Bocaina Formation microfossils in thin section. (**a**–**c**) Elongate, narrow, slightly curved, solid mineralized structures (ES). Thin sections GP5T/2555 (**a**,**c**) and 3FR21-26.35-26.52 (**b**). (**d**–**k**) Elongate, filled, tapered specimens (EF), described as EF1—well preserved walls (**d**–**f**) and EF2, steinkerns, lacking wall (**g**–**k**). Category EF displaying significant expansion toward one extremity with rounded termination (**d**,**h**–**k**), significant curvature and attenuation at one extremity (**e**–**g**,**j**), constriction near the narrower extremity pointed by red arrow (**h**,**k**) and random redirection of growth and sinuous profile along the length of the specimen (**h**,**j**,**k**). Thin sections GP5T/2555 (**d**–**f**), 3FR21-26.35-26.52 (**g**,**j**), GP5T/2570 (**h**-**i**), 3FR21-25.32-25.47x (**k**). (**l**–**q**). Equidimensional, circular to subcircular sections, filled by matrix (EQ1), displaying variable wall thickness and discrete indentations (grooves?) (red arrows in (**o**,**p**)). Thin sections GP5T/2555 (**i**), 18FR21-42.55 (**m**), 3FR21-25.41–25.58 (**n**,**q**), 3FR21-26.66-26.7 (**o**), 18FR21-42-42.28x (**p**). (**r**–**t**) Equilateral triangular structures (EQ2) with slightly convex sides and rounded vertices (**r**), planar sides and rounded vertices (**s**), or rounded vertices (lobes?) (**t**). Thin sections 3FR21-25.32-25.47x (**r**), 3FR21-25.32–25.47 (**s**), and 18FR21-42.55-42.9X (**t**). (**u**) Equidimensional, circular section, filled by phosphatized matrix (EQ1), displaying a thin wall, detailed in (**v**). Thin section FR-21b. (**w**–**x**) Elongate filled structures with coiled ends (CE) in thin section, with quasi-symmetrical longitudinal profile with an apparent medial constriction and incipiently convoluted walls (red arrows in (**w**)) and medially constricted quasi-symmetrical sheet-like structure with remarkably convoluted termini of walls at open end (**x**). Thin section GP5T/2570.

**Figure 8:**
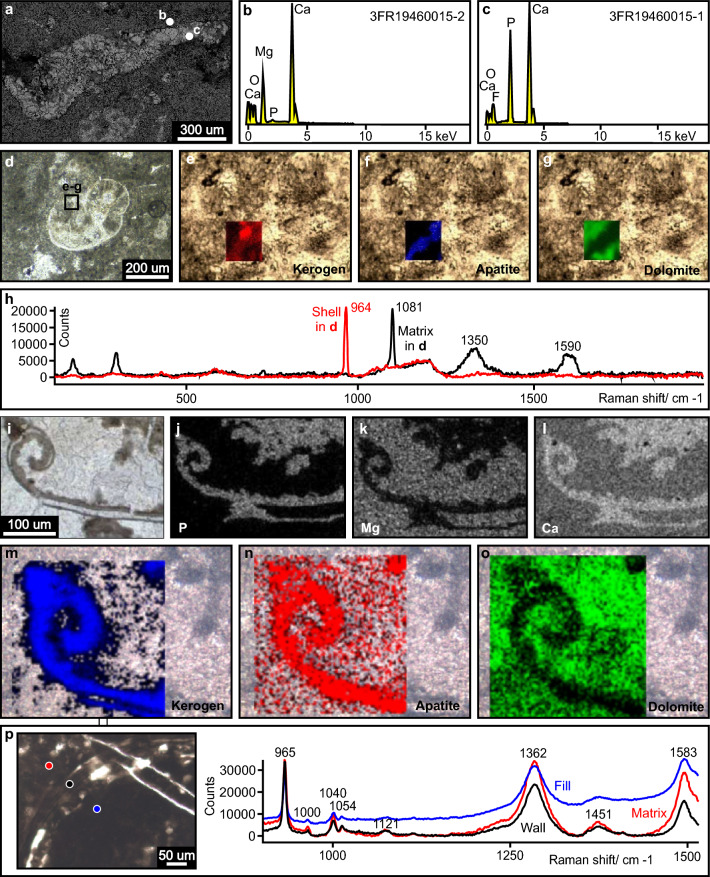
Examples of microfossil composition. (**a**) SEM image of ES specimen from the same sample that provided the thin section GP5T/2555. (**b**) Elemental point analysis spectrum of the host rock shown in (**a**), highlighting the presence of Ca and Mg and absence of P, consistent with dolomite. (**c**) Elemental spectrum from point analysis located in the microfossil wall shown in (**a**), show dominance of Ca and P and absence of Mg, consistent with apatite. (**d**) EQ2 specimen under transmitted light. Black square indicates the area investigated by Raman spectroscopy. Thin section GP5T/2555. (**e**–**g**) Digital Raman spectroscopy maps, showing diffuse concentration of kerogen in red (**e**), high concentration of apatite in blue (**f**), and matrix dominated by dolomite in green (**g**). (**h**) Comparison of Raman point analysis spectra, with high concentration of apatite (964 cm^–1^) in the wall of the specimen shown in (**d**), and occurrence of dolomite (1081 cm^–1^) and kerogen (1350 cm^–1^ and 1590 cm^–1^, band D and G, respectively) in the matrix. (**i**) Detail of coiled wall in a CE specimen, thin section GP5T/2570. (**j**–**l**) EDS maps of curled terminus of wall in (**i**). *P* phosphorus; *Mg* magnesium; *Ca* calcium. (**m**–**o**) Raman maps of kerogen, apatite and dolomite, respectively, of the same area. (**p**) Photomicrograph of EQ1 specimen (thin section FR-21b), displaying thin walls, similar to specimens in category CE, and respective Raman point analysis spectra showing homogeneous composition in the specimen and the host rock.

**Figure 9:**
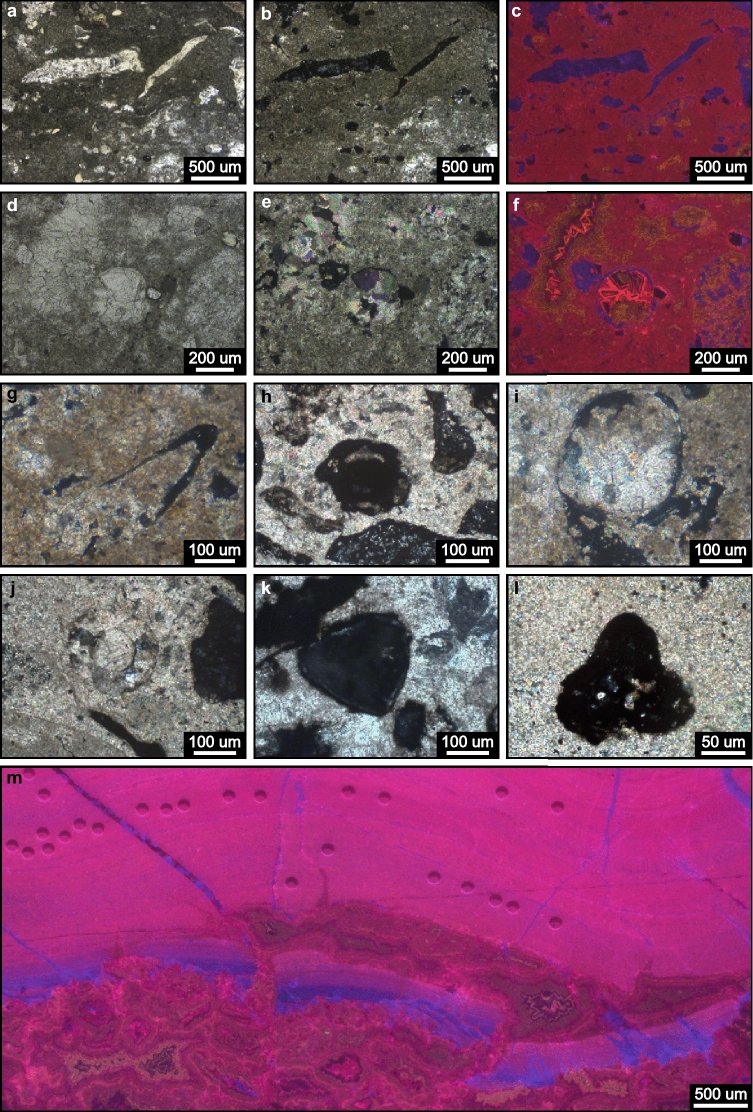
Petrographic features of bioclasts and associated microfacies. (**a**–**f**) Transmitted light (**a,d**), cross-polarized light (**b,e**) and CL images (**c,f**) of bioclasts and related microfacies from the Bocaina Formation. (**a**–**c**) EF2 (left) and ES (right) phosphatic specimens, as well as dispersed phosphatic fragments of likely bioclastic origin (purple in **c**) in dolomitic matrix (red in **c**) displaying incipient irregular lamination (**a,b**). (**d**–**f**) EQ1 specimen (near the center) displaying phosphatized wall (purple in **f**) with local dissolution and coarse carbonate cement fill (yellowish and pinkish CL colors in **f**). Dispersed phosphatized fragments and coarse cements in vugs are also observed within the dolomitic matrix (red in **f**) (**g**–**l**) Cross-polarized light images of bioclasts. (**g**) EF1 specimen with phosphatic wall and carbonate matrix fill. (**h**–**j**) EQ1 specimens (near the center of images) displaying variable wall thickness enclosing interior parts filled by matrix (**h**), matrix and cement in geopetal structure (see also Fig. 7l) (**i**) and cement (**j**). (**k**–**l**) Cross-polarized light images of EQ2 specimens preserved as phosphatic casts within fine dolospar. Note thin irregular carbonate relicts around the specimen in **k**. (**m**) CL image of fragmented stromatolite displaying concentrated and dispersed (very fine grained purple dots) phosphate within dolomitic microbial laminae. Note purple CL colors evidencing phosphate in vugs near the bottom of image and in fractures. Thin sections GP5T/2555 (**a**–**g**,**i**), 3FR21-26.66-26.7 (**h**), 18FR21-42-42.28x (**j**), 3FR21-25.32-25.47x (**k**), 18FR21-42.55-42.9x (**l**).

**Figure 10:**
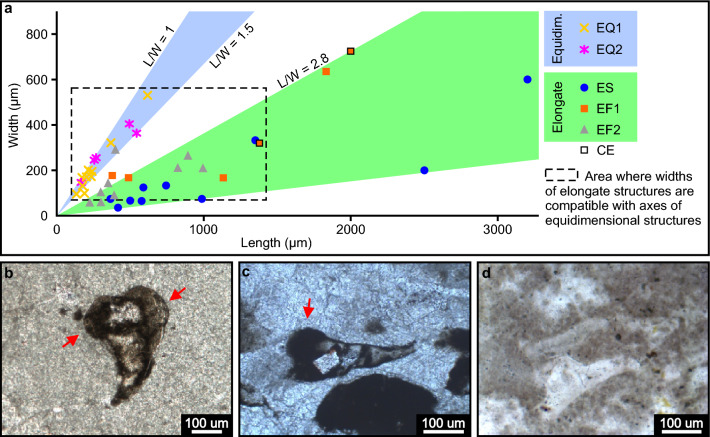
Connecting different microfossil cross sections to theoretical 3D shapes. (**a**) Scatter plot of lengths versus widths of the diverse microfossil morphotypes found in the Bocaina Formation. (**b**,**c**) Elongate specimens with expanded lobate apex interpreted as possible oblique cuts of tubular forms displaying transversal lobate tri-radial symmetry. Thin section 3FR21-26.35-26.52 (**b**) and 3FR21-25.32-25.47x (**c**). (**d**) Elongate specimen with thick walls probably due to near longitudinal tangential cut of tubular form. Thin section GP5T/2555.

The new fossil sites of the Tamengo Formation occur at coordinates 19° 32′ 50.96″ S, 57° 27′ 29.42″ W, between Porto Morrinhos and Puga Hill (Point SM01); 20º 32′ 37.86″ S, 56º 44′ 24.29″ W, ~ 6.5 km W from Bodoquena in the direction of Morraria do Sul (Point 22BD21); and 22º 13′ 59.31″ S, 56º 47′ 45.62″ W, ~ 4 km straight to SE from Caracol, (Point AP01E) (Figs. 1b, 2a,b). Regarding the studied Bocaina Formation fossil record (Figs. 7, 8, 9 and 10, Supplementary data, Tables 5, 6), we examined 69 samples from one-quarter of the drill cores ALR2-DD003, ALR2-DD004 and ALR2-DD018 and 11 samples from an open pit approximately 1.5 km north of the drill sites and belonging to the phosphate mining company EDEM (Empresa de Desenvolvimento em Mineração e Participações Ltda.) in the Serra da Bodoquena (Fig. 1b). In the studied phosphate mining area, bedding planes dip between 30º and 60º approximately to the east (Figs. 2b,d) and the drill cores ALR2-DD003, ALR2-DD004, and ALR2-DD018 were obtained through inclined drilling, approximately 60º to the west, aiming at a reliable representation of the true stratigraphic thickness of the surveyed deposits in the drill cores (Fig. 3).

Petrographic thin and thick sections, respectively ~ 40 and ~ 160 μm thick, were made from the samples collected in the open pit and along the drill cores. Thin sections and samples investigated under scanning electron microscope were used for microfossil analysis (morphology and composition) (Figs. 7, 8, 9 and 10). Thick sections were used for in situ U–Pb LA-ICP-MS geochronological analyses (Figs. 5, 6).

### Microfossil description

Morphometric features were obtained from petrographic thin sections using a Leica DM750 P microscope equipped with a Leica MC 170 HD camera (Supplementary data, Table 5). Morphology and composition were also analyzed using a LEO 440 scanning electron microscope (SEM), coupled with an energy dispersive spectrometer (EDS), at the Center for Geochronological Research (CPGeo-USP). Magnifications up to 200,000 × were obtained during the investigation, and structures as small as 1 µm were resolvable. Elemental (P, Mg, Ca) maps and spectra were created for the specimens (Fig. 8).

### Microfossil composition

Complementary investigation of microfossil compositions was carried out at the Brazilian Astrobiology Research Unit (NAP/Astrobio) in the Instituto de Química at the Universidade de São Paulo, using a Renishaw inVia micro-Raman, with 532 nm excitation wavelength, with step size of 0.7 µm, diode laser (500 mW) and 2400-groove/mm grating. A 20 × and 50 × objectives at 10% laser power was employed during 10 accumulations to collect spectra and to obtain Raman-mapping images for kerogen bands D and G (from 1130 to 1240 cm^–1^ and 1570 to 1647 cm^–1^), apatite (from 941 to 945 cm^–1^) and dolomite (from 1080 to 1107 cm^–1^) (Fig. 8).

Cathodoluminescence (CL) imaging was done at the Laboratory of Cathodoluminescence, Institute of Geosciences at the Universidade Federal do Pará, using a CITL Cathodoluminescence Mk5-2 equipment coupled to a petrographic microscope, with exposure time of 15.2 s, acceleration voltage of 352 to 520 A, and current of 15.5 to 16.0 kV (Fig. 9).

### U–Pb carbonate dating

Geochronological data were obtained along the studied succession via in-situ LA-ICP-MS dating of carbonate thick sections (~ 160 μm thick) at the Institute of Geochemistry and Petrology, ETH Zürich. A RESOlution S-155, 193 nm excimer (ArF) laser ablation system with a two-volume Laurin Technic ablation cell was coupled to a Thermo Element XR sector-field ICP-MS, following Guillong et al.^64^. Ablation with 110 μm, an energy density of 2 J cm^–2^ and a repetition rates of 5 Hz is under Helium atmosphere and the aerosol is mixed with argon and small amounts of nitrogen within the ablation funnel. The aerosole is finally homogenized by a squid smoothing device, and transported into the plasma of the Thermo Element XR ICP-MS. Detailed parameters are in the supplementary data (Table 1) along with the dataset with complete results (Table 2) and validation reference material results (Tables 3, 4).

Fine-grained microbialitic features and a vug observed in three thick sections (3FR19-35, 3FR19-43 and 4FR19-177) of predominantly dolomitic samples of the Bocaina Formation from two nearby cores (ALR2-DD003 and ALR2-DD004, Figs. 2c,d, 3, 5) were analyzed to determine uranium/lead (U/Pb) radioisotopic ratios. Eight isochrons were produced from 40 to 44 spot analyses, totaling 327 spots. Lower intercept ages were calculated using IsoplotR (Vermeesch^65^) with data plotted in Tera-Wasserburg diagrams (Fig. 6). Systematic uncertainties were propagated using excess uncertainty value of 2.5%^64^.

CL images of dated microfacies (Fig. 5) were obtained using a cold cathode cathodoluminescence (CITL CL) system (CL8200-model Mk5-2) at the Geological Institute of ETHZ. The CITL CL system was operated with an acceleration voltage of 15 kV, a beam current ranging from 0.1 mA to 0.5 mA under a vacuum of 0.03 mbar, and a current density from 5 to 10 μA/mm^2^.

## Results

### Updated correlation of the Corumbá Group Ediacaran carbonate successions across the South Paraguay Belt

The carbonate successions of the Bocaina and Tamengo formations of the Corumbá Group have been recognized and mapped across the South Paraguay Belt since the nineteenth century^66–72^. Recent advances in chemostratigraphy have corroborated the correlation of these units across the South Paraguay Belt, from the north around the Corumbá urban area, to the south, near the Brazil-Paraguay border^56,60,61^. However, abundant multicellular fossils, including the latest Ediacaran index-fossil *Cloudina*, are known at the northernmost and southernmost extremities of the South Paraguay Belt-Itapucumi Group setting, separated by approximately 380 km. In the Tamengo Formation in the area of Corumbá and Ladário (Figs. 1b, 2a), *Cloudina* is found mainly associated with microbialites^57,58^ and *Corumbella*^19,21, 58^. In Paraguay (Fig. 1b), *Cloudina* occurs with microbialites, *Corumbella* and *Namacalathus* in the Tagatiya Guazú Formation, Itapucumi Group^59,54, 60^. Here we extend the geographical occurrence of cloudinomorphs within the Tamengo Formation to three new sites (Figs. 1b, 2a,b), reducing the gap between known *Cloudina*-biozone occurrences in Central South America and reinforcing the correlation of the Ediacaran carbonate successions of the South Paraguay Belt.

Cloudinomorph specimens at these new localities consist of millimetric elongated sections expanding towards the apertural end, and display parallel constrictions along calcite walls (Fig. 4a–c). Tubular fossils constructed by nested, repeating, collared cylindrical units—commonly referred to as ‘funnel-in-funnel’ structures^73^—Can be locally observed (Fig. 4c). The northernmost new occurrence is from a bioclastic limestone bed directly above the contact with dolostones of the Bocaina Formation, more than 50 km to the southeast of the nearest fossiliferous outcrops of the Tamengo Formation in the Laginha quarry, Corumbá (Figs. 1b, 2a). The bioclastic limestone is dominated by cloudinomorph fragments, locally displaying a U-shaped section with constrictions (Fig. 4a). About 130 km southeast of this new locality, in Serra da Bodoquena (Figs. 1b, 2b), longitudinal cross-sections of cloudinomorphs are observed on bedding planes (Fig. 4b) in preserved pods within deformed limestone. Approximately 190 km south of the second new site (Fig. 1b), well-preserved specimens of *Cloudina* exhibiting typical nested (‘funnel-in-funnel’), multi-layered tubular construction (Fig. 4c)^74^ were recovered from limestone outcrops near the Rio Apa.

The new cloudinomorph occurrences reinforce the correlation of the uppermost Ediacaran section of the Corumbá Group across the South Paraguay Belt and Paraguay (Figs. 1b, 2b, c). In the studied phosphate mining area, we recognized the main geological contacts, confirming previous descriptions of their unconformable character (e.g. Refs.^43,44, 47,55^) (Fig. 2b). The Ediacaran carbonate deposits of the South Paraguay Belt (i.e. the Bocaina and Tamengo formations), as well as the Puga and Cerradinho formations mixed coarse siliciclastics and finely laminated carbonate deposits (e.g. Refs.^44,47^), occur in a slightly asymmetrical, west verging syncline, bounded to the west and east by inverse faults (Fig. 2b). The normal limbs of the synclinal structure and its parasitic folds can be readily recognized from base and top indications of stromatolitic structures (Fig. S1) and cross-stratified facies. Our new field data (as well as our geochronological results, see below) integrated with previously published results show that the new Bocaina Formation fossil record occurs underneath, and separated by a regional erosional unconformity from, the terminal Ediacaran biozone present in the Tamengo and Tagatiya Guazú formations.

### Carbonate U-Pb LA-ICP-MS geochronology

In situ U-Pb LA-ICP-MS analyses of carbonate samples from the fossiliferous interval, interpreted as microbialitic reef deposits, were performed aiming to geochronologicaly constrain the new fossil occurrences of the Bocaina Formation (Fig. 3). Homogeneous, fine-grained microfacies devoid of fractures and associated with stromatolitic laminae and thrombolites, as well as early cements in a vug (Fig. 5), provided eight isochrons with significant spread of ^238^U/^206^Pb ratios and relatively low mean square weighted deviation (MSWD) (Fig. 6). These eight isochrons, constructed from around 40 spot analyses each, provided calculated ages between 605 ± 28 and 559 ± 62 Ma, displaying MSWD values equal or below 2.2 and a weighted mean age of the 8 lower intercepts of 571 ± 9 Ma (8/8, MSWD = 1.1) (Fig. 6).

Four of the eight isochrons were obtained from the same petrographic features—two from adjacent areas in a thrombolite (Figs. 5b,c, 6a,b) and two from within the same vug (Figs. 5d,e, 6c,d). Each of these pairs pooled together provided calculated ages of 570 ± 11|18 Ma (n = 81, MSWD = 1.3) and 570 ± 16|21 Ma (n = 81, MSWD = 1.5), respectively (Fig. 6i,j). Uncertainties are given as 2σ and as propagated systematic uncertainties using the excess uncertainty value of 2.5%^64^. Ages with propagated systematic uncertainties were used to calculate the weighted mean age. These results reinforce the interpretation that the weighted mean age of 571 ± 9 Ma (Fig. 6k) is representative of the depositional and penecontemporaneous events that resulted in the fossiliferous record here described for the Bocaina Formation (Figs. 2, 3, 7).

### New microfossil assemblage

Objects interpreted here as microfossils come from phosphate-rich horizons associated with impure carbonate breccia, wackestone, packstone and grainstone, observed in the studied cores. These facies were also observed in the open pit mine, where diverse and thick microbialitic deposits, including meter-scale stromatolitic domes, are exposed and interpreted as shallow-water microbialitic reef facies association (Figs. 2d, 3; S1; Supplementary data, Tables 5, 6). Reef facies are intercalated with peritidal deposits, evidenced by impure fine-grained dolostone associated with thin dominantly stratiform microbialites, putative pseudomorphs after evaporite minerals (crystals with square outlines, needles, blades and nodular mosaics), rip-up clasts and soft-sediment deformation features (Fig. S2). The peritidal deposits transition upward into cross-laminated fine-grained siliciclastics (Figs. 2d, 3) that represent either progradation of continental input or an abrupt change in both subsidence rate and bathymetry.

Solid and filled objects hosted in the reef facies, observed in photomicrographs and electron micrographs of petrographic thin sections and hand samples, are here interpreted as sections of microfossil remains, shelled or not (Fig. 7). Based on the thin section studies, we separate these into five morphological categories as follows: elongate solid (ES) and elongate filled (EF) structures, characterized by length to width ratios ≥ 2.8; two categories of equidimensional structures (EQ1, EQ2), characterized by length to width ratios between 1.0 and 1.5; and elongate structures with coiled ends (CE) (Supplementary data, Table 5).

Category ES (N = 10): Slender, slightly curved, elongate solid objects, 365 to 3200 µm long and 37 to 600 µm wide, with length to width ratios between 4.1 and 13.2 (Supplementary data, Table 5), generally tapering at one end. One surface is flat to concave, generally smooth, and the other is convex, smooth or undulated (Figs. 7a–c, 8a, 9a–c). They are usually phosphate rich, locally made up of randomly oriented, inequigranular subhedral apatite crystals tens of microns in size (Figs. 8a,c, 9a–c).

Category EF (N = 13): Elongate gently tapering structures filled by matrix and/or cement, with (EF1) or without (EF2) preserved walls (Figs. 7d–k, 9g, 10b–d), some curving more or less abruptly near the narrow apex (Figs. 7e–g,j, 10b). Lateral margins can be parallel to slightly divergent (Figs. 7d–g,i, 9g, 10d), irregularly undulate (Figs. 7h,j,k, 10b,c), and constricted near the narrow apex (Fig. 7h,k). Length from 226 to 1833 µm, width 54 to 636 µm and length to width ratios from 2.9 to 6.8 (Supplementary data, Table 5). Two specimens present lobe-like thickenings at the expanded end (Fig. 10b,c). Walls 17 to 91 µm thick are preserved in four specimens (EF1) (Figs. 7d–f, 9g, 10d; Supplementary data, Table 5). These are usually P-rich (Fig. 9g).

Category EQ1 (N = 9): Circular to subcircular sections (Figs. 7l–q,u,v, 9d–f,h–j), filled by matrix and/or cement, displaying length to width ratios between 1.1 and 1.4, with longer axes from 138 to 620 µm and shorter axes 96 to 530 µm (Supplementary data, Table 5). EQ1 specimens are observed as more or less well-defined P-rich relict textures in fine (5–10 µm) sparry dolomite groundmass (Figs. 7l–q,u,v, 9d–f, h–j). Carbonate relicts are locally preserved (Fig. 9h,i). Wall thickness varies from 10 to 51 µm (Supplementary data, Table 5). One specimen displays a uniformly thick (15 µm), smooth wall made up of organic matter and phosphate, similar to the walls in category CE (Figs. 7u,v, 8p).

Category EQ2 (N = 4): Tri-radially symmetrical filled objects ranging from subtriangular (Fig. 7r), rounded triangular (Fig. 7s) to nearly trilobate (Fig. 7t), varying from 167 to 545 along major axes by 144 to 404 along minor axes, with length to width ratios between 1.0 and 1.5 (Supplementary data, Table 5). Walls, 11 to 18 µm thick (Supplementary data, Table 5), are poorly preserved as more or less well-defined P-rich relict textures in fine (5–10 µm) sparry dolomite groundmass with local carbonate relicts (Figs. 7r–t, 9k,l).

Category CE (N = 2): Seemingly bilaterally symmetrical, relatively large, elongate, sections with medial constriction and uniformly thick (10–30 µm), smooth walls (Fig. 7w,x) composed of kerogen and apatite (Fig. 8i–o). Amphora-like specimen in Fig. 7x is 2000 µm long and 725 µm wide and open at one end, where the walls curl tightly inward. The other specimen (Fig. 7w) is smaller, 1380 µm long and 320 µm wide, closed, rounded at one end and pointed at the other. Both are now filled by phosphatized matrix and crystalline dolomite (Fig. 8j–o).

### Fossil preservation

Microfossils reported here occur within P-rich horizons observed near the top of the microbialitic mound association, below the shaly peritidal facies association (Figs. 2d, 3; Supplementary data, Table 6). Silica-rich intervals, unrelated with Al, occur near the base and the top of the interval characterized by the P-rich horizons (Fig. 3). The microfossils often occur in a dolomicritic matrix, commonly recrystallized to fine dolomitic spar as observed under cross-polarized light (Fig. 9b,e,g–l) and indicated by EDS (Fig. 8a,b,k,l) and Raman analyses (Fig. 8g,h,o,p). Phosphorus-rich matrix is locally observed (Fig. 8j,n,p).

Microfossil wall composition is dominated by apatite, observed as very fine isotropic material under cross-polarized light (Fig. 9b,e,g–l), detected in Raman analyses (Fig. 8d,f,h,n,p) and indicated by high P and Ca in EDS analyses (Fig. 8a,c,j,l). Carbonate relicts of microfossil walls can be locally identified under cross-polarized light (Fig. 9e,h,i,k). SEM reveals local preservation of fossil wall as relatively coarse (dozens of microns), subhedral-to-euhedral, randomly oriented apatite crystals (Fig. 8a,c).

Microfossil infillings are dominated by matrix composed of dolomite and apatite, as observed under cross-polarized light (Fig. 9e,g-l) and indicated in EDS analyses (Fig. 8i–o). Relatively coarse sparry carbonate crystals are also observed filling microfossils (Fig. 9d,e), locally forming geopetal structures (Figs. 7l, 9i).

CL images show dull luminescent red fine dolomicritic matrix, P-rich bioclasts displaying purple colors, and vugs displaying carbonate cement formed by triangular and rhombohedral crystals (Fig. 9c,f), locally displaying P-rich purple coatings (Fig. 9m). These carbonate crystals have a zoned luminescence, with non-luminescent and bright yellowish zones that grade to zoned yellowish crystals (Fig. 9c,f). Purple P-rich fractures (Figs. 5i, 9m) and disseminated phosphate are also observed (Fig. 9m). P-rich bioclasts with dark purple luminescence are coated by irregularly distributed coarser, brightly luminescent, pinkish red carbonate crystals (Fig. 9f). Some bioclasts have an irregular outer margin (Fig. 9c) and some are infilled by zoned yellowish carbonate cements (Fig. 9f).

## Discussions and conclusions

### Interpretation of the Bocaina assemblage

The structures described above exhibit diverse outlines reminiscent of cross-sections of known skeletal microfossils and biogenic structures that are distinct from associated inorganic sedimentary particles. Each category includes several specimens and all occur as primary constituents of rocks clearly representing habitable depositional environments, as evidenced by the abundance of associated microbialites. Members of the five categories co-occur in all but one of the eight fossiliferous horizons found here (Supplementary data—Table 6). Moreover, widths of elongate structures match the sizes of equidimensional structures (Fig. 10a), supporting the interpretation that these cross-sections may represent, respectively, longitudinal and transverse cuts of tubular fossils, either cylindrical or conical. Intermediate length to width ratio values, between 1.5 and 2.8, and proportionally large wall thickness (Fig. 10b–d; Supplementary data, Table 5) are indicative of oblique and tangential cuts of these tubular objects. Similar forms lacking walls are interpreted as internal casts or steinkerns (Fig. 7g–k; Fig. 10b,c). For these reasons, we interpret them as microfossils.

### Biomineralization and diagenesis

The occurrence of abundant P-rich fragments reminiscent of biogenic structures with commonly preserved inner cavities filled by matrix and/or cement indicate that the studied microfossils were primary components. The phosphatic composition of the bioclasts could be primary or diagenetic. However, the occurrence of carbonate relicts within P-rich fossil walls (Fig. 9h,i,k) indicates a probable carbonate precursor. Phosphorus-rich coatings in the center of vugs, as well as phosphate in fractures and disseminated in matrix material (Figs. 5i, 9m), corroborate the hypothesis of diagenetic remobilization of phosphate resulting in phosphatization of originally carbonate bioclasts. The preferential preservation of the studied microfossils within a P-rich interval associated with silicified horizons below a permeable barrier (Fig. 3), also converges with this hypothesis. Signs of dissolution in P-rich bioclasts (Fig. 9a–f) and occurrence of steinkerns (Figs. 7g–k; 10b,c) evidence later partial to complete dissolution of phosphatized fossil walls.

Therefore, considering (i) the dominance of carbonate primary composition over phosphate and silica in marine fossil shells^10^, (ii) original calcareous composition of Ediacaran^21,75^ and Cambrian^10,75^ metazoan biomineralizers, (iii ) the phosphate adsorption power of carbonate shells^76–78^, (iv) the preferential precipitation of phosphate within inner shell microenvironments^79^; and (v) local preservation of carbonate walls, we suggest that the initial composition of the studied microfossils was likely carbonate, later replaced by phosphate.

Variable thickness along preserved walls and common occurrence as fragments indicate that the objects classified in the ES, EF1 and EQ morphotypes were originally rigid and therefore likely biomineralized remains. On the other hand, the uniformly thin, parallel, and smooth walls with coiled extremities in category CE suggest that these fossil remains were flexible and non-biomineralized.

### Comparison with Neoproterozoic and Cambrian fossils

Categories ES, EF and EQ invite comparison with cross sections of remains of diverse small skeletal elements of metazoans known from the Neoproterozoic and Early Cambrian^24,80^, which include: (i) Late Ediacaran to early Cambrian millimetric to centimetric calcareous tubular cloudinomorphs of the Nama biota^20,81^ and (ii) Fortunian Stage to Stage 3 millimetric small shelly fossils^25,79^. Category CE invite comparison with: (i) microscopic, phosphatized multilamellar, roll-up structures associated with microbial mats^82^, (ii) coiled organic sheets of the problematic Ediacaran to Cambrian small carbonaceous fossil Cochleatina^1^ and (iii) Early Cambrian carbonaceous sheets interpreted as surficial coverings of animals^8^.

The elongate cross-sections observed here may represent random cuts through platy structures or approximately longitudinal to tangential sections through hollow or solid cylindrical, conical, or ellipsoidal objects, be they open or closed, straight or slightly expanding, curved or not. Similarly, equidimensional cross-sections may correspond to ideal transverse cuts through bodies like those mentioned above or to diameters or small circles of spheroids.

This is particularly relevant when considering that the two specimens with thin walls and coiled extremities (CE) in the same thin section in Fig. 7w,   x and the circular cross-section (EQ1) in Fig. 7u,v, from the same sample (Supplementary data—Table 6), could represent different cross sections of the same thing. To wit, the two long sections in Fig. 7w,   x are similar in shape, despite the larger being open and the smaller closed. The diameter of the EQ1 specimen in Fig. 7u (530 by 620 µm) is similar to the width of the larger CE specimen (725 µm), and the walls in all three specimens are essentially identical in thickness (15 to 20 µm), structure, and organo-phosphatic composition (Fig. 8i–p). Once categories are separated based on the morphology in thin section, the specimen in Fig. 7u fits in category EQ1 while specimens in Fig. 7w,x fits in category CE. The uniformity and single-sheet construction of their walls and their apparent bilateral symmetry distinguish them from (i) roll-up biofilm microfacies associated with microbial mats, characterized by bifurcating and anastomosing laminae entangling detrital grains^82^ and (ii) the Ediacaran to Cambrian small carbonaceous fossil Cochleatina, characterized by coiled spiral-shaped ribbon ornamented with fine serrations on the surface^83^. Category CE could be more easily compared to Early Cambrian carbonaceous sheets^8^. These fossils are described as regular and thin sheets with sharp boundaries on both faces and squared-off ends. They exhibit sinuous folds, commonly enrolled to form a tight spiral, interpreted as surficial coverings of animals^8^. Category CE shares numerous similarities with these Early Cambrian carbonaceous sheets. Although lacking tight spirals and squared-off ends, they are here interpreted as putative flexible coverings (e.g. sclerite) of metazoans.

The other objects described here resemble remains of biomineralized organisms from the Late Ediacaran and Early Cambrian, such as cloudinomorphs and small shelly fossils. However, bidimensional sections hinder the visualization of diagnostic structures that can only be observed in tridimensional view, hampering the identification of their biological affinity. In addition, these fossils occur in rocks dated here as 571 ± 9 Ma, older than the appearance of cloudinomorphs in the Nama Biota, and older than the diversification of small shelly fossils in the Fortunian Biota.

The solid elongate specimens (ES) in Figs. 7a–c, 8a and 9a–c may represent random sections through plate- or scale-like sclerites or, alternatively, longitudinal or tangential sections of curved, tapering cylindrical or conical microfossils. Some share external outline, curvature and size comparable with the expected longitudinal cross sections of the earliest Cambrian protoconodonts, such as *Protohertzina*, as illustrated by Ref.^84^, their Fig. 3l–m). However, it was not possible to directly observe diagnostic features of this genus, such as lamellar wall structure and central cavity, in the ES specimens, although some thick walled EQ1 specimens could represent transverse cuts of elongate elements displaying a central cavity. Additionally, known protoconodonts display phosphatic composition interpreted as primary, whereas the phosphatic composition of all specimens in ES is here interpreted as related to late diagenetic phosphatization.

The elongate filled specimens (EF) expanding slightly upwards with thick wall profiles in Figs. 7d–f and 10d resemble typical longitudinal cross-sections of several biomineralized tubular Ediacaran fossils, such as cloudinomorphs^73,81^, but lack multilayered walls or “funnel-in-funnel” construction typical of cloudinids^81^. The circular to subcircular cross-sections of category EQ1 (Fig. 7l–q) could represent transverse cross-sections through such tubes.

The subtriangular to trilobate cross-sections of category EQ2 are reminiscent of expected transversal sections of Cambrian anabaritids and hyolithids. Like expected longitudinal sections of the anabaritid *Cambrotubulus decurvatus*, some EF specimens exhibit a gentle expansion of the apertural end and a sharp inflection near the apex (Figs. 7g,   j, 10b)^80,85^. The triangular outlines of EQ2 in Fig. 7r–t evoke comparison with the tri-radial tubular *Anabarites trisulcatus*. The EF specimens with lobe-like apertural structures in Fig. 10b,c may represent oblique cuts through anabaritid shell. EF specimens with a constriction near the apex and an undulating profile are similar to expected longitudinal sections of the hyolithid *Microcornus* (Figs. 7h,   k) illustrated by Missarzhevskii^86^, Fig. 35, pg. 124). Finally, it should be noted that some equidimensional cross-sections (Fig. 7l–q) are comparable to expected transverse cross-sections of more than one group of tubular small shelly fossils, such as hyolithids and protoconodonts^24,80, 85^, in addition to cloudinomorphs ^81^. The EQ1 specimen in Figs. 7o exhibits nearly perfectly circular inner and outer outlines, the latter with a roughly crenulate surface that could represent longitudinal striae or subtle lobes observed in several anabaritids^22,28,80,84,85^.

### Implications of the Bocaina assemblage

Morphometric analysis of frequent cross-sections of diverse bioclasts in phosphatic carbonates of the Bocaina Formation (Corumbá Group, Southern Paraguay Belt) dated at 571 ± 9 Ma (U–Pb weighted mean age) suggests that four of five morphological categories were produced by early metazoans able to biomineralize, whereas one may represent the flexible cuticle of an early metazoan. This diversified fossil assemblage thus predates the skeletal fossils of the uppermost Ediacaran *Cloudina* Interval zone (550 to 538.8 Ma) in the overlying Tamengo Formation, as well as the diversification and ascension of small shelly fossils in the Early Cambrian (Fortunian Stage through Stage 3; 538.8 to 514 Ma).

The Bocaina skeletal microfossils could be compared to expected longitudinal cross sections of several Ediacaran and Cambrian fossils, such as cloudinids, anabaritids, hyolithids and protoconodonts. However, we hesitate to assign them to formal taxa for two reasons: first, the temporal gap of at least tens of millions of years between these assemblages, i.e. 571 ± 9 Ma for the Bocaina Formation and the arguably latest Ediacaran occurrence of small shelly fossils^24^ and second, in view of the limits of the use of cross-sections for three-dimensional reconstructions adopted here. On the other hand, our results converge with the mid-Ediacaran onset of biomineralization estimated by molecular clocks^3^ and the early to mid Ediacaran diversified microfossils in the Doushantuo-Pertatataka assemblage^17^. Furthermore, some microfossils here described are reminiscent of Cambrian small shelly fossils occurring in uppermost Ediacaran strata, such as *Anabarites* e *Cambrotubulus* from the Siberian Platform^87^ and *Anabarites sp.* from the Dengying Formation in South China^20^. Thus, the forms described here may be interpreted as early expressions of the metazoan blueprint for biomineralization, long before the successful exploitation of this trait by small shelly fossils and cloudinomorphs^22,25, 81,88^. This reinforces previous suggestions that the Ediacaran and the Cambrian skeletal biota represents a continuum in the evolution of biomineralization, thus undermining the hypothesis of an Ediacaran-Cambrian extinction of skeletal organisms^20,87^. The co-occurrence of uni-^46^ and multicellular fossils resembling known Ediacaran and Cambrian forms in the Bocaina Formation, predating the occurrence of the latest Ediacaran index fossils in the Tamengo Formation, suggest that the Proterozoic-Phanerozoic fossil record may be strongly affected by local  environmental controls as well as sampling and taphonomic biases. As such, then, the Bocaina assemblage offers a unique and unexpected window into the evolution of biomineralization amongst early metazoans, revealing that they could have been developed and preserved diachronically in different parts of the globe.

### Supplementary Information

Supplementary Figure S1.

Supplementary Figure S2.

Supplementary Information.

Supplementary Legends.
