# Supplementary figures and images for: Dawn of diverse shelled and carbonaceous animal microfossils at ~ 571 Ma
Source: Sci Rep. 2024 Jun 28;14:14916. doi: 10.1038/s41598-024-65671-4 (PMC11213954; doi:10.1038/s41598-024-65671-4)

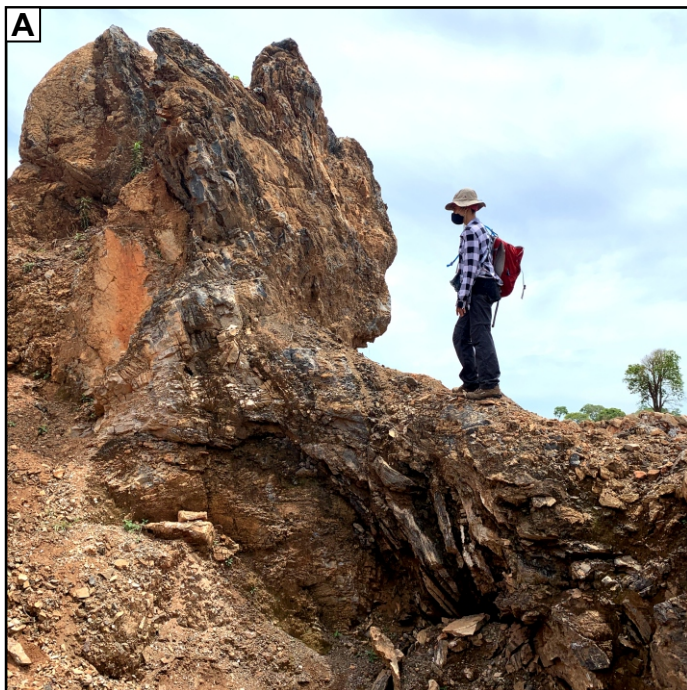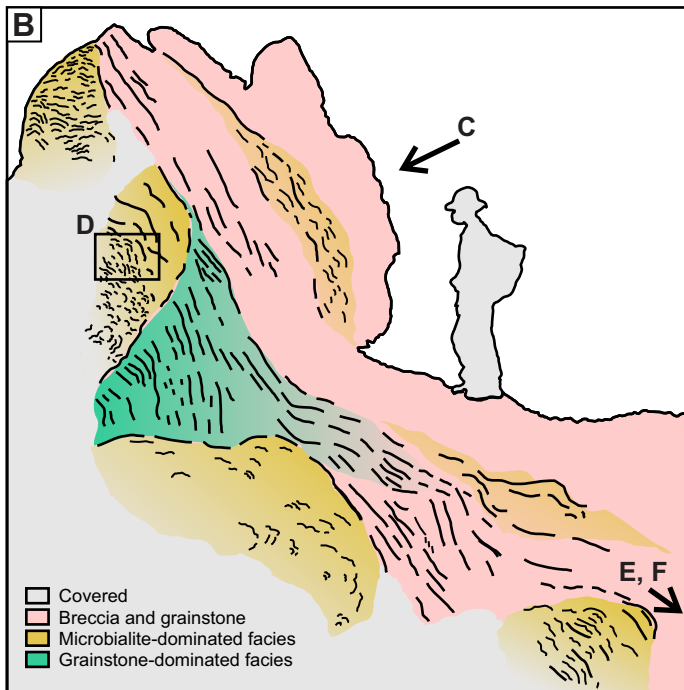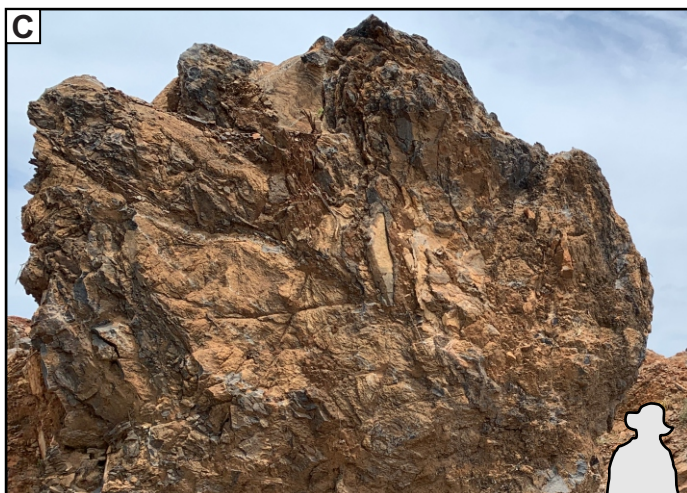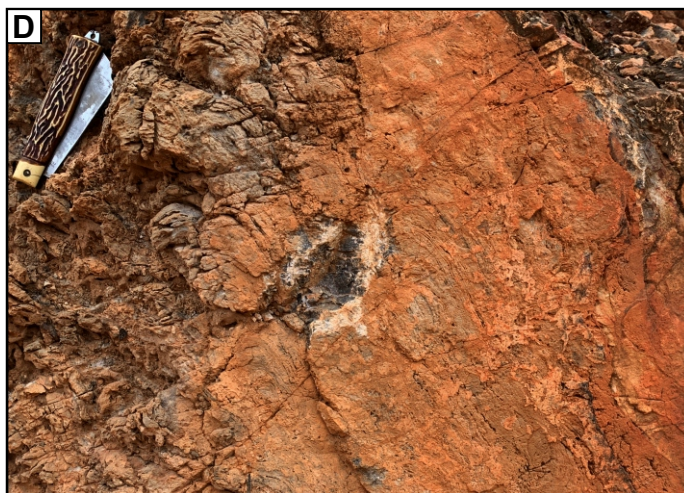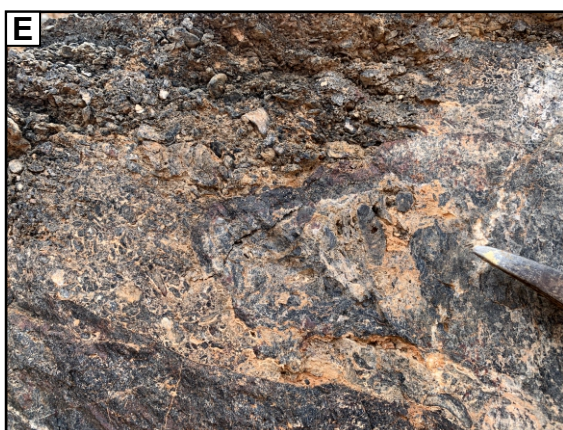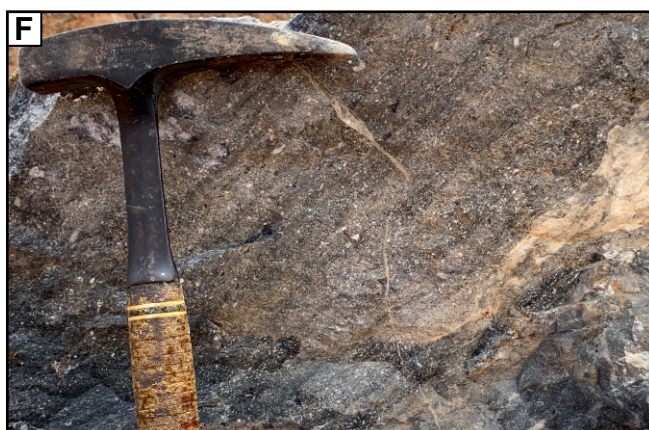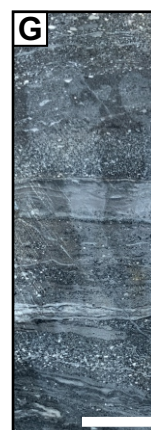

Supplement: Supplementary file 1 — Supplementary Figure S1. [file 41598_2024_65671_MOESM1_ESM.pdf]

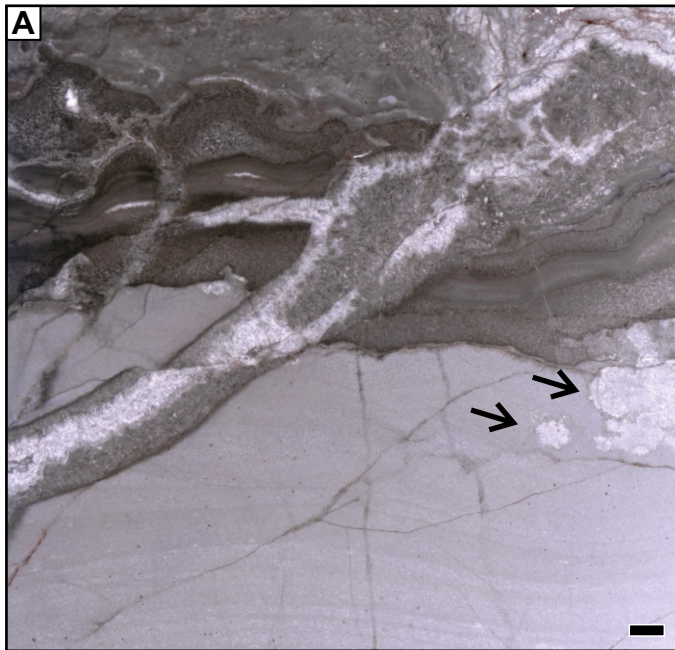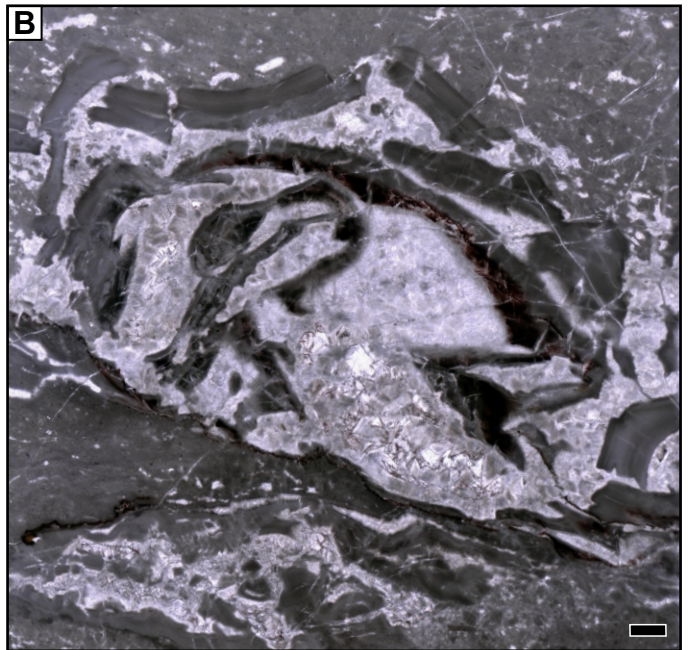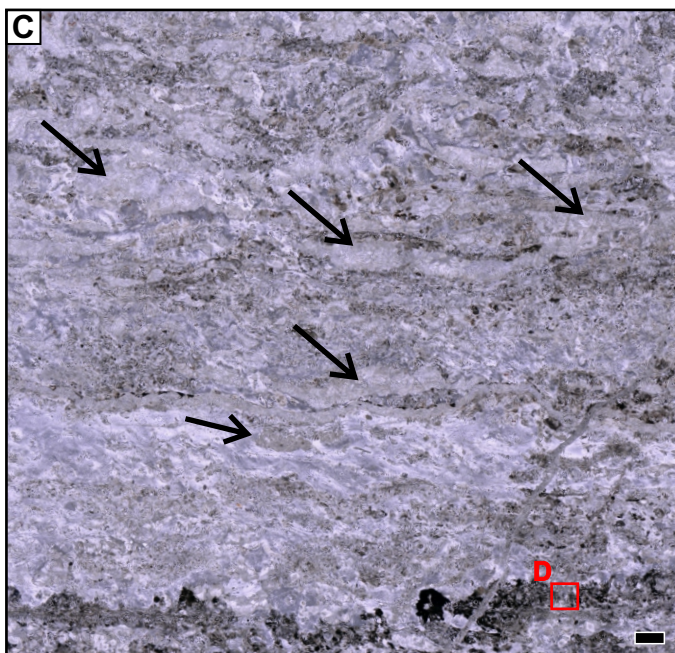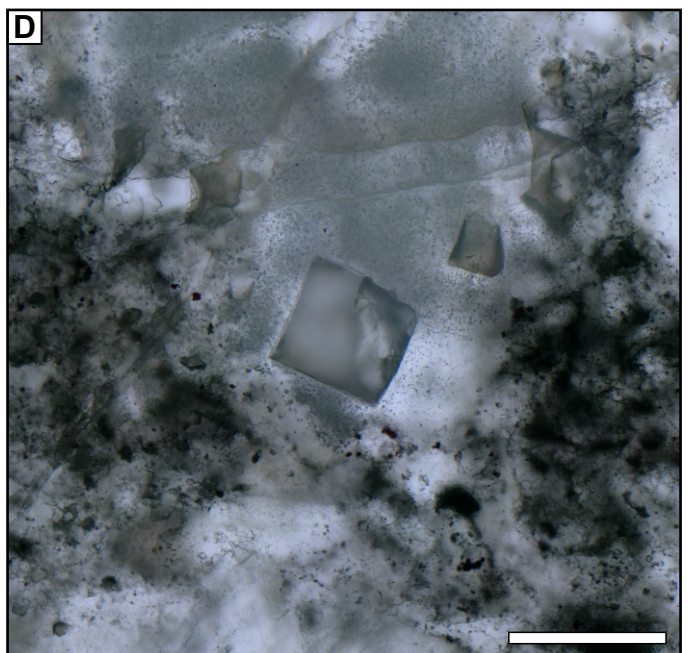

Supplement: Supplementary file 2 — Supplementary Figure S2. [file 41598_2024_65671_MOESM2_ESM.pdf]
