# Supplementary material for: Dawn of diverse shelled and carbonaceous animal microfossils at ~ 571 Ma
Source: Sci Rep. 2024 Jun 28;14:14916. doi: 10.1038/s41598-024-65671-4 (PMC11213954; doi:10.1038/s41598-024-65671-4)
Supplement: Supplementary file 4 — Supplementary Legends. [file 41598_2024_65671_MOESM4_ESM.docx]

Supplementary Figure Captions - ***Dawn of diverse shelled and carbonaceous animal microfossils at ~571 Ma***

Fig. S1. Examples of sedimentary features in the stromatolitic reef facies association. A. Cross-section view of tilted bedding in the EDEM phosphate mining area (20°49’55”S / 56°35’36”W) with graphical interpretation (B) showing relationships between microbialite boundstone, poorly-sorted grainstone and intraclastic breccia. C. Plan view of beds dominated by breccia and grainstone facies with minor intercalated microbialites. D. Detail of inclined microbialite bioherm. Top towards upper right. Pocket knife, upper left, is 11 cm long. E. Detail of poorly sorted coarse-grained facies composed of rounded to angular phosphorite intraclasts. F. Poorly sorted, sand-grade, fossiliferous grainstone facies. G. Drill core displaying the same facies as in F with minor microbialite intercalations. Scale bar is 2.5 cm.

Fig. S2. Examples of interpreted evaporitic features in the peritidal facies association of the Bocaina Formation. A. Microbial boundstone with irregular nodules displaying rims of small crystal blades (black arrows) interpreted as evaporite pseudomorphs. B. Curved and broken lamina sets associated with a pocket of nodular mosaic and crystalline cement interpreted as produced by volume changes and inter-stratal migration of salt within microbial mats in an evaporitic environment. C. Nodular mosaics (black arrows), evaporite pseudomorphs, within microbial boundstone. D. Close-up of a silicified portion of C showing a square section of an individual crystal interpreted as halite and a crystal arrangement to the upper right resembling halite hoppers. Black scale bars = 1 mm. White scale bar = 0.2 mm.
